# Association between Job-Related Factors and Work-Related Anxiety, and Moderating Effect of Decision-Making Authority in Korean Wageworkers: A Cross-Sectional Study

**DOI:** 10.3390/ijerph18115755

**Published:** 2021-05-27

**Authors:** Sang-Woo Kim, Junghee Ha, June-Hee Lee, Jin-Ha Yoon

**Affiliations:** 1 Department of Occupational and Environmental Medicine, Soonchunhyang University Hospital, Seoul 04401, Korea; 125155@schmc.ac.kr; 2 Department of Psychiatry, Institute of Behavioral Science in Medicine, Yonsei University College of Medicine, Seoul 03722, Korea; JH2672@yuhs.ac; 3 The Institute for Occupational Health, Yonsei University College of Medicine, Seoul 03722, Korea; 4 Department of Preventive Medicine, Yonsei University College of Medicine, Seoul 03722, Korea

**Keywords:** work-related anxiety, job-related factors, decision-making authority, wageworkers

## Abstract

Among the factors causing workers’ anxiety, job-related factors are important since they can be managed. Therefore, this study aimed to analyze the association between work-related anxiety and job-related factors among Korean wageworkers using data from the Fifth Korean Working Conditions Survey. Participants were 13,600 Korean wageworkers aged <65 years. We analyzed the association between job-related factors and work-related anxiety, and the moderating effect of decision-making authority. “Meeting precise quality standards,” “Solving unforeseen problems on your own,” “Complex tasks,” “Learning new things,” “Working at very high speed,” and “Working to tight deadlines” were positively associated with work-related anxiety. “Monotonous tasks” was negatively associated with work-related anxiety. The odds ratio (OR) of “Complex tasks” was higher in the group that had insufficient decision-making authority (OR 3.92, 95% confidential interval (CI) 2.40–6.42) compared to that with sufficient decision-making authority (OR 2.74, 95% CI 1.61–4.67). The risk of work-related anxiety was higher when the workers experienced time pressure, carried out tasks with high mental and physical demands, and dealt with unpredictable situations. This association was more pronounced when decision-making authority was insufficient.

## 1. Introduction

The negative effects of jobs on workers’ mental health include job-related stress, anxiety, depression, and burnout syndrome, and these not only reduce the productivity of the company but also affect the physical and mental health of the individual workers [1]. According to a report by the Health and Safety Executive, workers’ mental health problems are associated with frequent absenteeism, turnover, and early retirement [2]. Melchior et al. [3] reported that the risk of mental illnesses, such as major depressive disorder or anxiety disorder, was almost doubled when workers were exposed to high levels of job-related stress. In addition, job-related stress is also associated with physical disorders such as repetitive strain injury and work-related upper limb disorders [4].

Recently, various studies on the improvement of workers’ mental health have been conducted, but most have focused on job-related stress and major depressive disorder. Anxiety is the most common mental problem encountered in primary care, along with depression [5,6]. Anxiety and anxiety-related disorders negatively affect not only workers’ mental health but also their job performance [7,8]. Bronisch and Wittchen [9] reported that the risk of suicide attempt was significantly higher in individuals with both major depressive disorder and anxiety disorder than in those with only major depressive disorder. In a previous study on medical workers, anxiety was associated with burnout syndrome and had a negative effect on long-term absenteeism and job performance [10,11]. These studies suggest the need to manage workers’ anxiety along with job-related stress and depression to improve their mental health.

In previous studies that examined the risk factors of anxiety, physiological factors such as cognitive impairment, vision or hearing impairment, high blood pressure, neuroticism, dependent personality disorder, and past psychiatric history have been suggested [12,13,14,15,16,17]. As social factors, job-related factors such as lack of social support and job-related stress have been emphasized [12,18,19]. However, these studies have been conducted mainly in Western countries since the 1990s. A previous study that directly compared the Korean Working Conditions Survey (KWCS) and European Working Conditions Survey confirmed that Korean and European workers may show different results for the same factor due to cultural and environmental differences [20]. This is presumed to be due to differences in occupational safety and health-related regulations and systems, economic environment, and cultural differences in each country. The KWCS is the only Korean data that can directly compare the working environment of each European country with that of Korea using the same questionnaire. Therefore, this study aimed to analyze the association between job-related factors and work-related anxiety and determine the moderating effect of decision-making authority in Korean wageworkers using the fifth KWCS.

This study can provide basic information on what kind of job-related factors Korean wage workers feel anxious about. Workers’ mental health can be affected by various factors, including genetic predisposition as well as environmental and socio-psychological factors. Among these, job-related factors are important in that they can be managed through the improvement of organizational management, workplace culture, and working environment. Therefore, identifying job-related factors that cause work-related anxiety may not only improve workers’ job satisfaction and productivity, but can also be used as basic data in the field of occupational health to improve workers’ mental health.

## 2. Materials and Methods

### 2.1. Study Participants

This study used the Fifth KWCS conducted in 2017 [21]. The KWCS is conducted by the Occupational Safety and Health Research Institute under the Safety and Health Agency. The data included information on overall work environment such as employment type, occupation, type of business, exposure to various risk factors, and employment security for employed persons over 15 years old.

The Fifth KWCS included 50,205 subjects. We selected wageworkers under the age of 65 with a working period of at least 1 year. Respondents who answered, “Not applicable,” “Don’t know/no opinion,” or “Refuse to answer” to the independent and dependent variables were excluded. Finally, 13,600 participants were included in the analysis.

### 2.2. Measures

#### 2.2.1. Dependent Variable

A self-report questionnaire was used to evaluate work-related anxiety. Anxiety was assessed with the question “Over the last 12 months, did you have any of the following health problems (Anxiety)?” For the workers who answered “Yes,” we further investigated whether the problem was related to work. Work-relatedness was assessed by the question “(If so) Was it caused by your job?” Those who answered “Yes” to both questions were defined as experiencing work-related anxiety.

#### 2.2.2. Independent Variables

Job-related factors were assessed with the question “Generally, does your main paid job involve A–F?” and “Generally, does your main paid job include the following situations?” (G and H). The detailed question specified “meeting precise quality standards” (A), “assessing the quality of your own work” (B), “solving unforeseen problems on your own” (C), “monotonous tasks” (D), “complex tasks” (E), “learning new things” (F), “working at very high speed” (G), and “working to tight deadlines” (H). For G and H, study subjects were classified based on exposure of more than 1/4 of working hours.

Decision-making authority was assessed with the question “Are you able to choose or change A–C?” The detailed question specified “your order of tasks” (A), “your methods of work” (B), and “your speed or rate of work” (C). We defined decision-making authority as sufficient when an employee had two or more of these.

#### 2.2.3. Other Covariates

Sociodemographic and occupational characteristics that could act as potential confounders in the analysis were included as covariates. The sociodemographic and occupational characteristics of the study participants were evaluated using a self-report questionnaire. Educational level was classified into elementary school, middle school, high school, and college, based on the highest level of education successfully completed. The number of working hours per week were classified into less than 40 h, 40–51 h, 52–59 h, and 60 h or more, based on the Labor Standards Act and the overwork standards of the Industrial Accident Compensation Insurance Act [22,23]. Employment status was classified into temporary, fixed-term, and permanent based on employment type and contract period. Monthly income (in million won) was classified into less than 100, 100–199, 200–299, 300–399, and 400 or more based on the net salary for the recent three months. The company size was classified into less than 5, 5–29, 30–299, and 300 or more employees based on the total number of workers in the head and branch office. In this study, we reclassified 10 occupational categories into five categories: managers, professionals, and technicians were classified as “upper white collar,” and office workers were classified as “white collar.” Service workers and sales workers were classified as “service and sales”; laborers were classified as “unskilled manual” and skilled agricultural/fishery workers, craft and related trades workers, and plant/machine operators and assemblers were classified as “skilled manual.”

### 2.3. Statistical Analysis

We calculated descriptive statistics and analyzed the association between work-related anxiety and sociodemographic and occupational characteristics of the study participants. Continuous variables were summarized as mean and standard deviation, and categorical variables were summarized as frequencies and percentages. The means of continuous variables were compared using the *t*-test, and the frequency of categorical variables was compared using Pearson’s chi-square test.

We examined the prevalence of work-related anxiety according to job-related factors using the chi-square test. We conducted a logistic regression analysis to examine the relationship between job-related factors and work-related anxiety. The analysis was conducted using three models considering sociodemographic and occupational variables. Model 1 involved univariate analyses. Demographic variables, namely age, gender, educational level, and monthly income, were included as covariates in Model 2. Occupational variables, namely employment status and occupational category, were additionally included as covariates in Model 3. The odds ratios (ORs) and 95% confidence intervals were calculated.

We analyzed the moderating effect of decision-making authority on the association between job-related factors and work-related anxiety. We classified the study participants into four groups according to job-related factors and decision-making authority. Group 1 reported the absence of job-related factors and sufficient decision-making authority. Group 2 reported the presence of job-related factors and sufficient decision-making authority. Group 3 reported the absence of job-related factors and insufficient decision-making authority, and group 4 reported the presence of job-related factors and insufficient decision-making authority. We calculated the ORs for work-related anxiety in each group. The logistic regression analysis was conducted adjusting for age, gender, education level, monthly income, working hours per week, job status, and occupational classification. A two-tailed significance level of 0.05 was used, and all data were analyzed using R 3.6.3 (The R foundation for Statistical Computing, Vienna, Austria).

## 3. Results

Among the 13,600 total study participants, 210 reported work-related anxiety over the past 12 months, accounting for 1.54% of the total. We compared the sociodemographic and occupational characteristics of the study participants according to work-related anxiety (Table 1). Company size was significantly associated with work-related anxiety. The prevalence of work-related anxiety was 0.99% for companies with fewer than five employees, 1.38% for companies with 5–29 employees, 2.02% for companies with 30–299 employees, and 2.22% for companies with 300 or more employees. Age, gender, educational level, monthly income, working hours, employment contract, and occupational category did not show significant associations with work-related anxiety.

Table 2 shows the prevalence of work-related anxiety according to job-related factors. The prevalence of work-related anxiety was significantly associated with the presence of the following factors: meeting precise quality standards, solving unforeseen problems on your own, complex tasks, learning new things, and working at very high speed. The prevalence of work-related anxiety according to the presence or absence of complex tasks was 2.33% and 1.00%, respectively, which was the highest prevalence. The prevalence of work-related anxiety in the group with monotonous tasks was 1.07%, which was lower than that in the group without monotonous tasks (1.89%).

We performed a logistic regression analysis to confirm the relationship between job-related factors and work-related anxiety (Table 3). Those reporting “meeting precise quality standards” (OR 1.38, 95% confidence interval [CI] 1.03–1.86), “solving unforeseen problems on your own” (OR 1.47, 95% CI 1.09–1.99), “complex tasks” (OR 2.79, 95% CI 2.05–3.81), “learning new things” (OR 1.84, 95% CI 1.38–2.46), “working at very high speed” (OR 1.60, 95% CI 1.18–2.16), “working to tight deadlines” (OR 1.55, 95% CI 1.16–2.08) were more likely to experience work-related anxiety. On the other hand, those reporting “monotonous tasks” showed a lower risk (OR 0.48, 95% CI 0.35–0.66) of work-related anxiety.

Table 4 shows the moderating effect of decision-making authority on the relationship between job-related factors and work-related anxiety. For workers reporting “complex tasks” and “monotonous tasks,” those who lacked decision-making authority showed significantly higher ORs (3.92, 95% CI 2.40–6.42; 0.60, 95% CI 0.38–0.93) than those who had sufficient decision-making authority (2.74, 95% CI 1.61–4.67; 0.42, 95% CI 0.24–0.72). For the rest of the job-related factors, the likelihood of work-related anxiety was higher in the group that lacked decision-making authority.

## 4. Discussion

Anxiety and depression are the most common mental problems encountered in primary care [18,24]. In Korea, major depressive disorder is included in the Mid-Life (Turning Point) Health Examination and is screened every 10 years for individuals over the age of 20 [25]. Conversely, the prevalence and management status of anxiety and anxiety-related disorders in the Korean population are not well understood. Generalized anxiety disorder, panic disorder, social anxiety disorder, and post-traumatic stress syndrome are included in the categories of DSM-V anxiety disorders [26,27,28,29]. Of the 965 patients visiting primary care clinics in the United States of America between 2004 and 2005, 19.5% had at least one anxiety disorder, but a large number of respondents (41%) were not currently receiving treatment [30].

Anxiety is an emotional state that is closely related to depression and job-related stress and burnout [10]; job-related factors associated with work-related anxiety can also be interpreted based on the job demand–control model [31], which is a model of job-related stress proposed by Karasek [32] in 1979. The job demand–control model is the most traditional and influential theory regarding the job and mental health of workers; it explains the causes of job-related stress, focusing on job structure and characteristics. This model analyzes the work environment that affects the mental health of workers considering two aspects: job demand and job control. Accordingly, job-related stress occurs when employees are exposed to a job structure with high job demands and low job control.

In the present study, job-related factors related to high job demands were associated with work-related anxiety. Job demands can be measured by factors such as workload, time pressure, role conflict, and physical and emotional demand [33,34]. Among the job-related factors, individuals “working at very high speed” and “working to tight deadlines” are likely to have high job demands in terms of time pressure. “Meeting precise quality standards” and “learning new things” can also be linked to high job demands in physical and emotional terms.

“Assessing the quality of your own work” and “solving unforeseen problems on your own” are related to job control. Job control can be expressed as decision latitude for work and is divided into two elements: skill discretion and decision authority [31]. The previous two job-related factors imply that the worker has some decision-making authority. According to the job demand–control model, giving workers decision-making authority has a positive effect on their mental health in terms of job control. In this study, “solving unforeseen problems on one’s own” was significantly associated with work-related anxiety. This can be interpreted based on unique cognitive characteristics of anxiety. On the cognitive side, anxiety is triggered by uncertainty regarding possible future threats [35]. In the general job demand–control model, decision-making authority is considered positive, as it allows workers to actively perform their job [32]. On the other hand, considering the cognitive aspect of anxiety, decision-making authority can cause anxiety to workers by imposing responsibility for uncertain problems that may arise in the future.

“Complex tasks” and “monotonous tasks” showed the strongest association with work-related anxiety in this study. The odds ratio for work-related anxiety among workers who performed complex tasks was 2.79 (95% CI 2.05–3.81), and in particular, for lack of decision-making authority, it was 3.92 (95% CI 2.40–6.42). The odds ratio for work-related anxiety among workers who performed monotonous tasks was 0.48 (95% CI 0.35–0.66), showing a negative association. Workers who perform complex tasks may have higher workloads and physical and mental demands than those performing monotonous tasks. In addition, workers who perform complex tasks are likely to have to deal with various unpredictable situations, and this uncertainty can increase their anxiety.

Decision-making authority provides workers with skill discretion. According to the buffer hypothesis of the job demand–control model, job control can reduce the negative effects of high job demands on mental health [33]. For the job-related factors associated with work-related anxiety, the risk of work-related anxiety was higher when decision-making authority was insufficient except for “complex tasks” and “monotonous tasks.” Regarding these two job-related factors, the group who lacked decision-making authority had a greater risk of work-related anxiety than the group who had sufficient decision-making authority. According to buffer theory, decision-making authority may play a role in buffering the negative impact of high job demands on work-related anxiety. This means that if sufficient decision-making authority is given to workers, the risk of work-related anxiety can be reduced. For a more accurate analysis, a follow-up study on the interaction between high job demands and decision-making authority is needed.

The limitations of this study are as follows. As work-related anxiety was evaluated using the questionnaire in the Fifth KWCS, the prevalence reported in this study may have been underestimated or overestimated. In addition, causal relationships could not be inferred because of the cross-sectional nature of the data. It seems appropriate to interpret that specific job-related factors cause anxiety. Nevertheless, a clear interpretation of cause-effect is needed through prospective research. The questionnaire used in the Fifth KWCS evaluated only the presence of anxiety; thus, detailed information about the intensity of anxiety and situations that cause anxiety was insufficient. Anxiety under appropriate circumstances is a normal emotional response and should be distinguished from anxiety disorder, a disorder requiring treatment. Although the Fifth KWCS data had limitations due to lack of information needed to assess the association with anxiety disorder, it seems to be meaningful in itself to reveal job-related factors that cause workers’ anxiety. In addition, in the KWCS, questionnaires about job-related factors consist of a single item response. Therefore, it is difficult to evaluate the degree of each job related-factor, and there is a limitation on the comprehensive evaluation social support, organizational culture, and resources. For more accurate analysis in the future, follow-up studies using standardized evaluation tools are needed.

The strengths of this study are as follows. Since it was based on the Fifth KWCS, which provided nationally representative data of Korean workers, it was possible to determine the percentage of workers who felt anxious about work. In addition, we were able to identify job-related factors and occupational groups that are more vulnerable to work-related anxiety, which can be used as basic data for the classification of risk groups in the management of workers’ mental health. The Fifth KWCS includes information on the overall work environment, including work type, employment type, occupation, industry type, exposure to risk factors, and employment security. The strength of this study is that it revealed the relationship between specific job-related factors and work-related anxiety, while most previous studies indirectly scored the level of job demand and control using evaluation tools.

In this study, we analyzed the risk factors of work-related anxiety based on the principles of the job demand-control model and using data from the Fifth KWCS. There was an increased risk of work-related anxiety for workers performing their tasks under high time pressure, with high job demand, and coping with unpredictable situations. Decision-making authority buffered the negative impact of job-related factors on work-related anxiety. Anxiety shares similar characteristics with general mental health problems, but there are also differences due to its inherent cognitive characteristics. Workers who perform tasks associated with work-related anxiety are at a higher risk of experiencing anxiety and need to be managed accordingly. In addition, in order to alleviate workers’ anxiety, it is necessary to provide sufficient decision-making authority regarding the order, method, and speed of their work. Currently, job-related factors are an important aspect of workers’ mental health problems. To address this, research focusing on vulnerable groups and job-related factors that can act as risk factors must be conducted. We trust that the results of our study can be used as basic data for managing workers’ anxiety and anxiety-related disorders.

## Figures and Tables

**Table 1:** Sociodemographic and occupational characteristics of study participants according to work-related anxiety.

	Work-Related Anxiety			
Variable	Yes	No	Total	*p*-Value
Total	210 (1.54%)	13,390 (98.46%)	13,600 (100%)	
Age				
Mean (SD)	44.72 (10.60)	44.28 (10.58)	44.29 (10.58)	0.546
Age category				
<30	16 (1.21%)	1303 (98.79%)	1319 (100%)	0.812
30–39	53 (1.51%)	3457 (98.49%)	3510 (100%)	
40–49	62 (1.54%)	3968 (98.46%)	4030 (100%)	
50–59	62 (1.70%)	3583 (98.30%)	3645 (100%)	
≥60	17 (1.55%)	1079 (98.45%)	1096 (100%)	
Sex				
Male	122 (1.74%)	6875 (98.26%)	6997 (100%)	0.061
Female	88 (1.33%)	6515 (98.67%)	6603 (100%)	
Educational level				
Elementary school	2 (1.25%)	158 (98.75%)	160 (100%)	0.596
Middle school	12 (2.03%)	580 (97.97%)	592 (100%)	
High school	80 (1.66%)	4749 (98.34%)	4829 (100%)	
College	116 (1.45%)	7895 (98.55%)	8011 (100%)	
Monthly income (million won/month)				
<100	6 (0.87%)	684 (99.13%)	690 (100%)	0.294
100–199	72 (1.51%)	4696 (98.49%)	4768 (100%)	
200–299	65 (1.47%)	4356 (98.53%)	4421 (100%)	
300–399	38 (1.70%)	2195 (98.30%)	2233 (100%)	
≥400	28 (2.05%)	1335 (97.95%)	1363 (100%)	
Working time (hours/week)				
<40	14 (1.34%)	1033 (98.66%)	1047 (100%)	0.492
40–51	159 (1.50%)	10,467 (98.50%)	10,626 (100%)	
52–59	12 (2.01%)	585 (97.99%)	597 (100%)	
≥60	25 (1.90%)	1290 (98.10%)	1315 (100%)	
Employment contract				
Temporary	16 (1.31%)	1204 (98.69%)	1220 (100%)	0.159
Fixed-term	13 (2.51%)	504 (97.49%)	517 (100%)	
Permanent	181 (1.53%)	11,682 (98.47%)	11,863 (100%)	
Company size (Number of employees)				
<5	27 (0.99%)	2711 (99.01%)	2738 (100%)	0.002
5–29	86 (1.38%)	6167 (98.62%)	6253 (100%)	
30–299	46 (2.02%)	2226 (97.98%)	2272 (100%)	
≥300	49 (2.22%)	2156 (97.78%)	2205 (100%)	
Occupational category				
Upper white collar	28 (1.18%)	2335 (98.82%)	2363 (100%)	0.268
White collar	52 (1.37%)	3740 (98.63%)	3792 (100%)	
Sales and service	66 (1.76%)	3687 (98.24%)	3753 (100%)	
Skilled manual	40 (1.64%)	2403 (98.36%)	2443 (100%)	
Unskilled manual	24 (1.92%)	1225 (98.08%)	1249 (100%)	

**Table 2:** Prevalence of work-related anxiety according to job-related factors.

	Work-Related Anxiety			
	Yes	No	Total	*p*-Value
Meeting precise quality standards				
Yes	91 (1.91%)	4655 (98.09%)	4746 (100.00%)	0.012
No	119 (1.34%)	8735 (98.66%)	8854 (100.00%)	
Assessing the quality of your work				
Yes	110 (1.58%)	6857 (98.42%)	6967 (100.00%)	0.784
No	100 (1.50%)	6533 (98.50%)	6633 (100.00%)	
Solving unforeseen problems on your own				
Yes	144 (1.79%)	7909 (98.21%)	8053 (100.00%)	0.007
No	66 (1.19%)	5481 (98.81%)	5547 (100.00%)	
Monotonous tasks				
Yes	61 (1.07%)	5648 (98.93%)	5709 (100.00%)	<0.001
No	149 (1.89%)	7742 (98.11%)	7891 (100.00%)	
Complex tasks				
Yes	129 (2.33%)	5404 (97.67%)	5533 (100.00%)	<0.001
No	81 (1.00%)	7986 (99.00%)	8067 (100.00%)	
Learning new things				
Yes	95 (2.19%)	4235 (97.81%)	4330 (100.00%)	<0.001
No	115 (1.24%)	9155 (98.76%)	9270 (100.00%)	
Working at very high speed				
Yes	148 (1.84%)	7885 (98.16%)	8033 (100.00%)	<0.001
No	62 (1.11%)	5505 (98.89%)	5567 (100.00%)	
Working to tight deadlines				
Yes	142 (1.83%)	7605 (98.17%)	7747 (100.00%)	0.002
No	68 (1.16%)	5785 (98.84%)	5853 (100.00%)	

**Table 3:** Association between job-related factors and work-related anxiety.

	Odds Ratio (95% Confidence Interval)		
	Model 1	Model 2	Model 3
Meeting precise quality standards	1.43 (1.09–1.89) *	1.37 (1.03–1.81) *	1.38 (1.03–1.86) *
Assessing the quality of your own work	1.05 (0.80–1.38)	1.02 (0.78–1.35)	1.02 (0.78–1.35)
Solving unforeseen problems on your own	1.51 (1.13–2.03) **	1.47 (1.10–1.98) *	1.47 (1.09–1.99) *
Monotonous tasks	0.56 (0.42–0.76) ***	0.52 (0.38–0.72) ***	0.48 (0.35–0.66) ***
Complex tasks	2.35 (1.78–3.11) ***	2.57 (1.90–3.47) ***	2.79 (2.05–3.81) ***
Learning new things	1.79 (1.36–2.35) ***	1.88 (1.41–2.49) ***	1.84 (1.38–2.46) ***
Working at very high speed	1.67 (1.24–2.25) ***	1.63 (1.21–2.20) **	1.60 (1.18–2.16) **
Working to tight deadlines	1.59 (1.19–2.13) **	1.55 (1.16–2.07) **	1.55 (1.16–2.08) **

*** *p* < 0.001 ** *p* < 0.01 *p < 0.05. Model 2: adjusted by age, gender, educational levels, monthly income. Model 3: adjusted by age, gender, educational levels, monthly income, employment contract, number of employees, occupational category.

**Table 4:** Moderating effect of decision-making authority on the association between job-related factors and work-related anxiety.

	Odds Ratio (95% Confidence Interval)			
	1	2	3	4
Meeting precise quality standards	1	1.49 (0.92–2.44)	1.36 (0.91–2.04)	1.88 (1.20–2.95) **
Assessing the quality of your own work	1	1.27 (0.75–2.16)	1.59 (0.91–2.45)	1.50 (0.90–2.49)
Solving unforeseen problems on your own	1	1.76 (0.92–3.38)	1.56 (0.81–2.99)	2.39 (1.27–4.51) **
Monotonous tasks	1	0.42 (0.24–0.72) **	1.13 (0.80–1.60)	0.60 (0.38–0.93) *
Complex tasks	1	2.74 (1.61–4.67) ***	1.33 (0.81–2.19)	3.92 (2.40–6.42) ***
Learning new things	1	1.42 (0.87–2.31)	1.13 (0.75–1.70)	2.68 (1.72–4.18) ***
Working at very high speed	1	1.36 (0.82–2.27)	1.07 (0.64–1.80)	1.85 (1.17–2.93) **
Working to tight deadlines	1	1.51 (0.91–2.52)	1.23 (0.74–2.05)	1.93 (1.22–3.07) **

*** *p* < 0.001 ** *p* < 0.01 * *p* < 0.05. 1: Decision-making authority “Sufficient,” job-related factors “No”. 2: Decision-making authority “Sufficient,” job-related factors “Yes”. 3: Decision-making authority “Lack,” job-related factors “No”. 4: Decision-making authority “Lack,” job-related factors “Yes”.

## Data Availability

The data described in this article are openly available at https://oshri.kosha.or.kr/oshri/researchField/workingEnvironmentSurvey.do (accessed on 10 March 2020).
